# Malignancy and chemotherapy induced haemophagocytic lymphohistiocytosis in children and adolescents—a single centre experience of 20 years

**DOI:** 10.1007/s00277-018-3254-4

**Published:** 2018-02-06

**Authors:** Volker Strenger, Gerald Merth, Herwig Lackner, Stephan W. Aberle, Harald H. Kessler, Markus G. Seidel, Wolfgang Schwinger, Daniela Sperl, Petra Sovinz, Anna Karastaneva, Martin Benesch, Christian Urban

**Affiliations:** 1 Department of Paediatrics and Adolescent Medicine, Medical University of Graz, Auenbruggerplatz 34/2, A-8036 Graz, Austria; 2 Department of Virology, Medical University of Vienna, Vienna, Austria; 3 Institute of Hygiene, Microbiology and Environmental Medicine, Medical University of Graz, Graz, Austria

**Keywords:** Haemophagocytic lymphohistiocytosis, Children, Procalcitonin, Viral reactivation

## Abstract

Haemophagocytic lymphohistiocytosis (HLH) is a possibly life-threatening syndrome of immune dysregulation and can be divided into primary (hereditary) and secondary forms (including malignancy-associated HLH (M-HLH)). We retrospectively analysed epidemiological, clinical, virological and laboratory data from patients with M-HLH treated at our department between 1995 and 2014. Out of 1.706 haemato-/oncologic patients treated at our department between 1995 and 2014, we identified 22 (1.29%) patients with secondary HLH (1.3–18.0, median 10.1 years; malignancy induced *n* = 2; chemotherapy induced *n* = 20). Patients with acute myeloblastic leukaemia (AML) developed HLH significantly more often than patients with acute lymphoblastic leukaemia (ALL) (10/55, 18.2% vs. 6/148, 4.1%, *p* = 0.0021). As possible viral triggers, we detected BKV (53.8% of the tested patients), HHV-6 (33.3%), EBV (27.8%), CMV (23.5%), ADV (16.7%) and PVB19 (16.7%) significantly more frequently than in haemato-/oncologic patients without HLH. Despite lacking evidence of concurrent bacterial infection, C-reactive protein (CRP) and procalcitotnin (PCT) were elevated in 94.7 and 77.7% of the patients, respectively. Ferritin and sIL2R were markedly elevated in all patients. HLH-associated mortality significantly (*p* = 0.0276) decreased from 66.6% (1995–2004) to 6.25% (2005–2014), suggesting improved diagnostic and therapeutic management. Awareness of HLH is important, and fever refractory to antibiotics should prompt to consider this diagnosis. Elevated ferritin and sIL2R seem to be good markers, while inflammatory markers like CRP and PCT are not useful to discriminate viral triggered HLH from severe bacterial infection. Re-/activation of several viruses may play a role as possible trigger.

## Introduction

Haemophagocytic lymphohistiocytosis (HLH) is a hyperinflammatory disorder characterised by immunologic dysregulation including activation of macrophages as well as cytotoxic T and natural killer (NK) cells leading to cytopenia, fever and hepatosplenomegaly as well as characteristic changes in laboratory parameters including elevated ferritin and soluble CD25 (interleukin-2 receptor). HLH can be divided into primary or hereditary forms and secondary forms. Primary or hereditary HLH is characterised by a variety of different genetically hetereogenous disorders (including Griscelli syndrome, familial HLH 2-5, X-linked lymphoproliferative syndrome) [1–3]. Onset of primary HLH is commonly observed during childhood, often triggered by a common infection. In contrast, secondary forms of HLH commonly occur during different kinds of immune alterations including malignancy, iatrogenic immunosuppression including cytotoxic chemotherapy and haematopoetic stem cell transplantation (HSCT), autoimmune diseases or infections. The latter has often been reported as an additional trigger during conditions with altered immune status. Most frequently described infectious agents triggering HLH are/include Epstein Barr virus (EBV), cytomegalovirus (CMV), human immunodeficiency virus (HIV), influenza A virus, *Mycobacteria* spp. and *Leishmania* [3–5]. Beside immunological factors, parenteral nutrition containing lipids is considered as a possible trigger of HLH [6, 7].

An important subgroup of secondary HLH is the malignancy-associated HLH (M-HLH) which can be divided into malignancy triggered forms, where the malignancy itself leads to HLH, and the chemotherapy-associated HLH, where the antineoplastic treatment or its side effects serve as a trigger of HLH [2, 8]. However, during treatment of malignancies, these two conditions might overlap and a clear etiologic discrimination might be difficult. Typical clinical symptoms of HLH may resemble symptoms of malignant diseases as well as severe bacterial infections often occurring during antineoplastic treatment and subsequent immunodeficiency [2, 3, 8, 9]. Chemotherapy-associated HLH in fact is often reported to be triggered by infections and the hyperinflammatory state seems to be the consequence of an inadequate reaction of the dysregulated immune system against common infectious agents. While symptoms may lead to the clinical diagnosis of an infection (with or without identifying the infectious agent), the subsequent immune dysregulation and inadequate immune activation might often remain unidentified leading to a high number of undiagnosed cases of HLH [10]. While Delavigne et al. recently described the detection of HLH in up to 10% of the adult acute myeloid leukaemia (AML) patients [11], incidence of malignancy-associated (either malignancy triggered or during chemotherapy) HLH in paediatric haemato-/oncologic patients is not known, so far. Furthermore, systematic data on possible infectious triggers, C-reactive protein (CRP) and procalcitotnin (PCT), two parameters often used as marker for bacterial infections, are scarce or even missing in patients with HLH.

The aim of this study was to report our single centre experience with M-HLH in order to describe incidence and clinical features of this entity including possible triggers and laboratory parameters.

## Patients and methods

After approval by the local ethic committee, we evaluated patients who were diagnosed as having HLH during their treatment at the Division of Paediatric Haematology/Oncology, Department of Paediatrics and Adolescent Medicine, Medical University of Graz, between 1995 and 2014.

In addition to patients already registered as being diagnosed with HLH in the institutional database, we screened our electronic laboratory system (data available since 2005) for elevated ferritin levels and retrospectively analysed the identified patients for the fulfilment of the HLH criteria at the time of hyperferritinaemia.

HLH was defined according to the HLH-2004 diagnostic criteria which require that at least five out of the eight criteria listed in Table 1 are met [2, 12]. Patients with primary/hereditary HLH were excluded from further analyses.

**Table 1:** HLH-2004 diagnostic criteria for secondary HLH. At least five out of the eight criteria are required for the diagnosis of HLH (According to Lehmberg et al. [2, 12])

Diagnostic criteria for secondary HLH (five out of the eight criteria fulfilled)
- Fever
- Splenomegaly
- Cytopenia (affecting two of three lineages in the peripheral blood)
Haemoglobin <9 g/dl (in infants < 4 weeks: haemoglobin < 100 g/l)
Platelets < 100 × 10^9/l
Neutrophils < 1.0 × 10^9/l
- Hypertriglyceridemia and/or hypofibrinogenemia
Fasting triglycerides ≥ 3.0 mmol/l (≥265 mg/dl)
Fibrinogen ≤ 1.5 g/l
- Hemophagocytosis in bone marrow or spleen or lymph nodes
- Low or absent NK cell activity (according to local laboratory reference)
- Ferritin > 500 mg/l
- Soluble CD25 (soluble IL-2 receptor) > 2400 U/ml

Analyses for infectious agents were performed according to the institutional standards either as part of our comprehensive routine screening measures which are commonly applied in high risk patients in our institution or was additionally intensified to identify an infectious source of the respective febrile illness. Microbiological diagnostics changed during the 20 years period. While blood cultures were routinely taken in febrile patients, viral PCR from blood, urine and mouthwash were increasingly performed in the analysed years. To describe the significance of viruses detected during HLH episodes, we calculated the rate of positivity of viral PCR (“detection rate”) for the most relevant viruses obtained in haemato-/oncologic patients without HLH from March 2001 through December 2010 and compared detection rate of these viruses in specimens from haemato-/oncologic patients without HLH with the incidence of viral (re-)activation concurrently with HLH.

Statistical analyses were performed by means of Mann-Whitney *U* test, Spearman correlation and Fisher’s exact test using SPSS for windows 21.

## Results

### Incidence and underlying diseases

In our department, 1.706 patients were treated for malignant and non-malignant haematological as well as for oncological diseases between 1995 and 2014. Out of these, we identified 22 patients (1.29%, median 10.1 years, range 1.3–18.0 years, male/female 10/12) who met the criteria for HLH. The frequency of patients identified as having HLH increased from 6/634 patients (0.95%) within the first 10-year period (1995–2004) to 16/1072 patients (1.49%) within the second 10-year period (2005–2014). However, this increase is not significant (*p* = 0.383).

In two patients, HLH was the first symptom of a mediastinal germ cell tumour and an anaplastic large cell lymphoma, respectively. In the remaining 20 patients, HLH occurred during haemato-/oncologic treatment (see Table 2). Underlying diseases were AML (*n* = 10), acute lymphoblastic leukaemia (ALL, *n* = 6), solid tumours (*n* = 4) and 1 lymphoma (for details, see Table 2). One patient had undergone alloSCT for globoid cell leukodystrophy (Krabbe disease).

**Table 2:** Details on patients, their underlying diseases, treatment protocols, HLH-specific treatment, possible triggers, peak levels of inflammatory markers and outcome of the HLH

Pat. nr.	Year of onset	Age at onset of HLH	Sex	Underlying disease	Treatment protocol	State at diagnosis of HLH	HLH-specific therapy	Outcome of HLH	Preceding treatment with LAmB	Preceding parenteral nutrition	Concurrent infections	Peak CRP level (mg/l)	Peak PCT level (ng/ml)
1	1995	3.9a	m	Pre-B-ALL	ALL REZ BFM 90	Maintenance therapy	IVIG	Died (1 day after diagnosis)	n.a.	n.a.	InflA	n.a.	n.a.
2	1997	17.1a	f	AML FAB M2	AML BFM 93	Maintenance therapy	IVIG, Dxm, VP-16	Died (47 days after diagnosis)	No	no	EBV, paranasal aspergillosis	104.7	n.a.
3	1997	1.5a	m	Medulloblastoma	HIT SKK 92	Tumour progression under treatment	Dxm., VP-16	Died (2 days after diagnosis)	n.a.	Yes	CMV	n.a.	n.a.
4	2001	17.1a	m	Mediastinal germ cell tumour	HLH therapy	HLH as initial presentation	IVIG, VP-16, Dxm, Daclizumab, CS-A	Died (23 days after diagnosis)	No	No	–	16.0	n.a.
5	2002	15.5a	m	Anaplastic large T cell lymphoma	ALCLl	HLH as initial presentation	G-CSF, Dxm, VP-16	Remission	No	No	PVB19	25.0	n.a.
6	2002	11.0a	f	C-ALL, second relapse	ALL REZ BFM Pilot 02; BMT	Day + 68 after MUD BMT, acute GvHD	VP-16, Dxm, Daclizumab	Remission	Yes	Yes	EBV, HHV6, pulmonary aspergillosis	n.a.	n.a.
7	2005	10.7a	f	AML, FAB M2	AML BFM 2004	Day + 24 after allogeneic HLA identical BMT, acute GvHD	Dxm	Remission	No	Yes	–	< 5.0	n.a.
8	2006	12.4a	f	Ewing sarcoma	EURO Ewing 99	Sixth VIDE	Dxm, infliximab	Remission	Yes	Yes	–	128	n.a.
9	2007	14.8a	f	AML FAB M2 (refractory)	RIC MUD alloPSCT	Day + 2 after RIC PSCT	Infliximab, daclizumab, methylprednisolone	Remission	Yes	Yes	EBV	236	n.a.
10	2008	16.8a	f	AML FAB M1	AML BFM 2004	1 + 2: maintenance therapy	1: Dxm, infliximab; 2: Dxm, rituximab, IVIG, infliximab	Died in second episode (132 days after first diagnosis)	Yes	No	1: ADV, BKV, HHV6, PVB19; 2: EBV	264	n.a.
11	2008	18.0	f	Mb. Krabbe	Second HaploPSCT	Day + 22 after 2nd haploSCT (graft rejection after the 1st SCT)	Dxm, infliximab	Remission	No	No	Cerebral toxoplasmosis	157.0	0.47
12	2008	7.5a	f	Secondary AML after treatment of embryonal rhabdomyosarcoma	CWS 2002, MUD SCT	Day + 44 after MUD SCT	Dxm, IVIG	Remission	Yes	Yes	BKV, HHV-6, PVB19	42.4	n.a.
13	2009	2.4a	f	AML FAB M6 refractory to treatment	Allogeneic peripheral SCT	Conditioning for allogeneic SCT	Dxm, infliximab	Remission	Yes	Yes	HHV6, RSV	220.4	13.59
14	2010	10.9a	f	AML FAB M5	AML BFM 2004 (HR)	1: AI-2CDA consolidation, 2–4: maintenance treatment	Dxm	Remission after fourth episode	Yes	Yes	1: BKV; 2:-4: CMV	90.2	n.a.
15	2011	1.3a	f	AML FAB M5a	AML BFM 2004	1: end of ADxE induction 2: re-induction of HAM	Dxm	Remission after second episode	Yes	Yes	1: EBV (weak: HHV 6, HHV7 and HHV8)	94.4	15.66
16	2011	17.4a	m	Osteosarcoma (relapse)	HaploSCT	Day + 8 after HaploSCT, graft rejection	Dxm	Remission	Yes	Yes	–	110.3	3.22
17	2012	7.3a	f	AML FAB M0/I	AML BFM 2004	End of ADxE induction	Dxm	Remission	n.a.	n.a.	–	317.5	12.4
18	2012	2.2a	m	AML FAB M5	AML BFM 2004	HAM protocol	Dxm	Remission	n.a.	Yes	HHV6, HSV1	53.1	13.57
19	2013	3.1a	m	T-ALL	AEIOP BFM ALL 2009	1: protocol IB-ASP 2: protocol III	Dxm	Remission after second episode	n.a.	n.a.	1: BKV; 2: CMV	37.4	1.8
20	2013	1.3a	m	Infant ALL	DLI/stem cell boost after 2nd HaploSCT	Day + 120 after haploSCT, day +12 after stem cell boost, acute GvHD	Dxm, sirolimus	Remission	Yes	Yes	JCV	190.3	1.08
21	2013	4.3a	m	T-ALL	AEIOP BFM ALL 2009	Protocol IB	Dxm, IVIG	Remission	No	Yes	ADV, BKV, HHV6	90.2	n.a.
22	2014	7.1a	m	Relapse of C-ALL	ALL REZ BFM	Day + 225 after alloBMT, chronic GvHD, colitis	Dxm	Remission	Yes	Yes	ADV, BKV, CMV	74.4	0.42

*m/f* male/female, *HR* high risk, *BFM* Berlin-Frankfurt-Münster, *MUD* matched unrelated donor, *BMT* bone marrow transplantation, *SCT* stem cell transplantation, *haplo* haploidentical, *RIC* reduced intensity conditioning, *VP-16* etoposid, *ATG* anti-thymocyte globulin, *DLI* donor lymphocyte infusion, *CS-A* cyclosporine A, *Dxm* dexamethasone, *n.a.* not available, *ADV* adenovirus, *BKV* BK virus, *CMV* cytomegalovirus, *EBV* Epstein-Barr virus, *HHV* human herpesvirus, *HSV* herpes simplex virus, *InflA* influenza A virus, *JCV* JC virus, *PVB19* parvovirus B19, *RSV* respiratory syncytial virus

≠ is not

Eight patients developed HLH after allogeneic stem cell transplantation (alloSCT, *n* = 7) or bone marrow transplantation (alloBMT, *n* = 1). Four patients developed HLH within 30 days after alloSCT/BMT, whereas intervals between SCT and occurrence of HLH in the other four patients were 44, 68, 120 and 225 days, respectively. One patient (pt. 16) had concurrent acute graft rejection, while three patients had graft versus host disease (GvHD) at the time of HLH.

With respect to the total number of patients treated for AML (*n* = 55) and ALL (*n* = 148) during the analysed period, there was a significant higher rate of patients with HLH in AML patients (10/55, 18.2%) than in ALL patients (6/148, 4.1%, *p* = 0.0021).

### Clinical symptoms

Fever was present in all 22 patients. Hepatomegaly and splenomegaly were documented in 17/22 (77.2%) and 16/22 (72.7%) patients, respectively. Pulmonologic symptoms were documented in 9/22 (40.9%) patients and comprised pleural effusion (*n* = 3), cough (*n* = 2), obstructive bronchitis (*n* = 3), pulmonary infiltrates (*n* = 1) and atelectases (*n* = 2). Dermatologic symptoms (exanthemas) were documented in 6/22 (27.3%) and comprised petechial bleedings and haematomas (*n* = 1) and maculopapular rashes (*n* = 5). Neurological symptoms were documented in 8/22 (36.4%) and comprised irritability (*n* = 1), cephalaea (*n* = 2), tremor (*n* = 1), spasticity (*n* = 1), seizures (*n* = 4), hallucinations (*n* = 1) and somnolence (*n* = 2). As a possible consequence of HLH, one patient (pt. 14) developed severe uveitis on both eyes with significantly reduced visus.

### Possible triggers

While 2 patients developed HLH as primary symptom of their disease (malignancy triggered HLH), 20 patients received cytotoxic chemotherapy prior to the onset of HLH (HLH during chemotherapy) including patients under maintenance therapy for AML (*n* = 2) or ALL (*n* = 1) and patients within 30 days after alloSCT (*n* = 5). Three patients had GvHD at the time of HLH.

Twenty-one of the 22 patients were analysed for viral infection or reactivation by PCR or RT-PCR for at least one virus. However, number of tested viruses differed between episodes. At the time of HLH, nucleic acid of at least 1 virus was identified in 16 (76.2%) of the tested 21 patients. In eight (50%) patients, more than one virus was identified within one episode of HLH (see Table 2) with up to four viruses simultaneously. Beside commonly described viral triggers of HLH like EBV (detected in 27.8% of the EBV tested patients), CMV (detected in 23.5% of the CMV tested patients) and ADV (detected in 16.7% of ADV tested patients), we found concurrent (re-)activation of BK virus (BKV, 53.8%), human herpesvirus 6 (HHV-6, 33.3%) and parvovirus B19 (PVB19, 16.7%), as well as human herpesvirus 7 (HHV-7), herpes simplex Typ 1 (HSV-1), influenza A, respiratory syncytial virus (RSV), human herpesvirus 8 (HHV-8) and JC virus (JCV) in single patients, each. Despite performing molecular diagnostics from blood, we did neither detect herpes simplex Typ 2 (HSV-2, tested in 13 patients) nor enteroviruses (tested in 17 patients). Detection rate in peripheral blood was significantly higher during HLH episodes for BKV, HHV-6, EBV, CMV, ADV and PVB19 when compared to detection rates in specimens from patients without HLH. For details on viral detection, see Table 3.

**Table 3:** Details on viral nucleic acid detection performed concurrently with HLH compared to detection in specimens from haemato-/oncologic patients without HLH. Results from blood specimens were analysed. Only for BKV, results from urine specimens were analysed additionally. BKV was only tested in blood specimens from four HLH patients with BK viruria and was positive in two (50%) of them. Detection rates of viruses detected concurrently with HLH only in one patient each were not compared (−)

Virus	Patients tested concurrently with HLH			Specimens tested in patients without HLH			
	Patients tested	Patients positive	Positive (% of tested)	Specimens tested	Specimens positive	Positive (% of tested)	*p* value
Commonly tested viruses							
BKV (urine)	13	7	53.8%	1.453	385	26.5%	0.051
HHV-6	18	6	33.3%	3.721	286	7.7%	0.002
BKV (blood)	7	2	28.6%	1.467	57	3.9%	0.029
EBV	18	5	27.8%	4.323	425	9.8%	0.027
CMV	17	4	23.5%	2.458	84	3.4%	0.002
ADV	18	3	16.7%	3.439	24	0.7%	< 0.001
PVB19	18	3	16.7%	3.631	148	4.1%	0.036
HHV-7	13	1	7.7%	–	–	–	–
HSV-1	15	1	6.7%	–	–	–	–
HSV-2	15	0	0.0%	–	–	–	–
VZV	17	0	0.0%	–	–	–	–
EV	17	0	0.0%	–	–	–	–
Occasionally tested viruses							
Influenza A	6	1	16.7%	–	–	–	–
RSV	7	1	14.3%	–	–	–	–
HHV-8	9	1	11.1%	–	–	–	–
JCV	9	1	11.1%	–	–	–	–
Influenza B	5	0	0.0%	–	–	–	–

*ADV* adenovirus, *BKV* BK virus, *CMV* cytomegalovirus, *EBV* Epstein-Barr virus, *EV* enterovirus, *HHV* human herpesvirus, *HSV* herpes simplex virus, *JCV* JC virus, *PVB19* parvovirus B19, *RSV* respiratory syncytial virus, *VZV* varicella zoster virus

In two patients with HLH, *Aspergillus fumigatus* infections of the nasal sinus (pt. 2) and the lungs (pt. 6), respectively, were documented. One patient (pt. 11) had concurrent cerebral toxoplasmosis. No bacterial infection was identified during HLH episodes despite multiple blood cultures obtained during febrile episodes.

At the time of (or immediately prior to) the development of HLH, parenteral nutrition was administered in 14/19 (73.7%) patients and liposomal formulation of amphotericin B (AmBisome®) was administered in 12/19 (63.2%) patients. In three patients, data on parenteral nutrition and liposomal amphotericin B were not available.

### Laboratory parameters

During HLH episodes, hyperferritinaemia, pancytopenia, increased soluble IL-2 receptor (sIL-2R) and hypoalbuminaemia were observed in 100%, hypertriglyceridaemia and hypofibrinogenaemia were observed in 17/18 (94.4%) and 7/18 (38.9%), respectively.

Although peak values of ferritin were 1 to 46.3-fold (median 3.22-fold) higher than the respective values at the onset of the episodes, ferritin at the onset of HLH was above the upper normal value in all patients (Table 4).

**Table 4:** Details on laboratory values during HLH

Laboratory test	Pathologic/patients tested (% pathologic)	Definition of pathologic values	Median value (range)
Pancytopenia	22/22 (100%)	at least 2/3 lineages in the peripheral blood affected*	**–**
Hypertriglyceridaemia	19/22 (86.3%)	>265 mg/dl (fasting)	351 mg/dl (142–1.099 mg/dl)
Hypofibrinogenaemia	7/18 (38.8%)	≤1.5 g/l	1.66 g/l (0.8–3.57 g/l)
Haemophagocytosis in BM. spleen or lymph nodes	14/15 (93.3%)	**–**	**–**
Initial hyperferritinaemia	17/17 (100%)	≥500 μg/l	4.757 μg/l (806–123.276 μg/l)
Peak hyperferritinaemia	17/17 (100%)	≥500 μg/l	11.802 μg/l (4.838–162.524 μg/l)
Elevation of CD25 (soluble IL-2-receptor)	18/18 (100%)	≥2.400 U/ml	6.255 U/ml (3.328–63.080 U/ml)
Elevation of procalcitonin	7/9 (77.7%)	≤0.5 ng/ml	3.22 ng/ml (0.42–15.66 ng/ml)
Hypoalbuminaemia	13/13 (100%)	≤3.5 g/dl	2.7 g/dl (2.1–3 g/dl)
Elevation of CRP	18/19 94.7%	≥8 mg/l	99.5 mg/l (<5–317.5 mg/l)

*BM* bone marrow, *CRP* C-reactive protein

*Anaemia (Hb < 9 g/dl). Thrombocytopenia (< 100 × 10^9/l). Granulocytopenia (< 1 × 10^9/l)

We additionally analysed inflammatory parameters commonly used for the diagnosis of infections. Without evidence of bacterial infections in any of the analysed episodes, CRP was elevated in 18/19 (94.7%) with values ranging from < 0.5 to 317.5 mg/l (median 99.5, normal <8 mg/l). PCT was elevated in 7/9 (77.7%) with values ranging from < 0.5 to 15.7 ng/ml (median 3.22, normal < 0.5 ng/ml). For details on CRP and PCT in different patients and different kinds of associated infections, see Table 2.

There were no statistically significant differences for any of the analysed parameters between fatal and non-fatal HLH cases. However, there were statistically significant correlations between peak values of ferritin and sIL-2R (*r* = 0.642, *p* = 0.010), ferritin and fibrinogen (negative correlation, *r* = −0.497, *p* = 0.036) as well as ferritin and CRP (*r* = 0.462, *p* = 0.046). Additionally, PCT peak values correlated significantly with the relative increase from initial to peak ferritin values (*r* = 0.543, *p* = 0.024).

### Treatment

Twenty-one patients received corticosteroids (dexamethasone, *n* = 20, methylprednisone, *n* = 1) either as a monotherapy (*n* = 5) or in combination with monoclonal antibodies against TNF-α (infliximab, *n* = 5), CD25 (daclizumab, *n* = 3) or CD20 (rituximab, *n* = 1) and with etoposide (*n* = 5), 7s immunoglobulins (*n* = 6) or rapamycin (*n* = 1). For details, see Table 2.

### Repeated episodes

In four patients, HLH reappeared after apparent resolution of signs and symptoms. One patient (pt. 10, AML FAB M1) developed HLH during AML maintenance therapy concurrently with (re-)activation of ADV, BKV, HHV-6 and PVB19. She recovered under dexamethasone and infliximab. Three months later, HLH re-appeared with EBV reactivation. The patient developed multi organ failure and died despite treatment with dexamethasone, infliximab, IVIG and rituximab as well as cidofovir and foscavir.

One patient (pt. 15, AML FAB M5a) developed two HLH episodes with only 1 month interval (at ADxE induction with reactivation EBV, HHV-6, -7 and -8 and at HAM re-induction, respectively), which both quickly responded to dexamethasone treatment.

One patient (pt. 19, T-ALL) developed two HLH episodes with an interval of 8 months (at protocol IB with BKV reactivation and at protocol III with CMV reactivation, respectively), which both quickly responded to dexamethasone treatment.

One patient (pt. 14, AML FAB M5) developed six HLH episodes (concurrently with HHV-6 and repeated CMV re-activations), which every time quickly responded, even though transiently, to dexamethasone treatment. However, AML maintenance therapy had to be stopped after only 2 months, leading to resolution of recurrent HLH. Despite early cessation of maintenance therapy, the patient is in first remission of AML 7 years after end of treatment.

### Outcome

While 17 patients recovered from HLH, 5 patients (22.7%) developed multi organ failure due to HLH and died despite intensified HLH-specific treatment (see Table 1). One of these had malignancy-induced HLH (pt. 4, mediastinal germ cell tumour), the remaining four patients developed fatal HLH during viral infection (pt. 1, Influenza A infection) or re-activation of EBV (pts. 2 and 10) or CMV (pt. 3), respectively.

While in the first half of the analysed period (1995–2004), four of six patients (66.6%) died due to HLH, only 1 of 16 patients (6.25%) died due to HLH in the second half (2005–2014, Fisher’s exact test *p* = 0.0276).

## Discussion

In this 20-year retrospective single centre analysis of malignancy-associated secondary HLH, we found an incidence of this condition of 1.2% of all paediatric haemato-/oncologic patients treated at our institution. HLH occurred significantly more often in AML patients than in ALL patients, which might be explained by the more aggressive treatment regimes. On the other hand, HLH also was observed during maintenance therapy for AML. However, M-HLH is not limited to patients with haematologic malignancies and was also seen in patients with solid tumours. While Lehmberg et al. described a series of 29 patients with malignancy-associated HLH in which the majority had a malignancy triggered (and not chemotherapy induced) form [8], in our series, only two patients initially presented with HLH prior to anti-neoplastic treatment.

While malignancy-induced HLH as well as HLH during chemotherapy are both summarised as malignancy-associated HLH, it should be highlighted that these two conditions are distinct entities. Chemotherapy-induced HLH might rather be the consequence of immune dysregulation during chemotherapy-induced myelosuppression than caused by the malignant disease itself. Thus, pathogenesis of chemotherapy-induced HLH differs from pathogenesis of malignancy-induced HLH. Therefore, we would recommend that chemotherapy-induced HLH should clearly be differentiated from malignancy-induced HLH and that these two different entities should not be summarised into the group of malignancy-associated HLH.

The diversity of HLH is also reflected by the variable clinical courses in our series. While some patients quickly responded to dexamethasone monotherapy, others showed severe complications like respiratory distress and seizures or developed multi organ failure and died despite escalating intensive treatment with corticosteroids, etoposide and different monoclonal antibodies. Furthermore, concomitant clinical symptoms varied widely and comprised partially severe pulmonologic and neurologic symptoms. However, some of the observed symptoms might also originate from other clinical conditions (e.g. exanthemas during GvHD). One patient developed severe uveitis leading to significant reduced visus. While HLH-associated uveitis has been previously reported in one case report [13], we cannot rule out other causes for this condition like CMV infection or cidofovir treatment.

Four patients developed repeated episodes of HLH. However, the end of HLH cannot be clearly defined. Therefore, recurrence in some of these patients might be interpreted as prolonged HLH with varying activity resembling relapses. In one of these patients (the patient who developed uveitis), HLH episodes repeatedly re-occurred over a period of 8 months every time an anti-leukaemic treatment was re-started until an AML maintenance therapy has been abandoned after only 2 months.

As recommended [2], treatment in M-HLH has to be decided case by case considering underlying diseases and their treatment, possible infectious triggers, which should be treated, and severity of HLH. Treatment modalities of M-HLH may follow the HLH-2004 protocol for non-malignancy-associated HLH but is not limited to agents used herein (corticosteroids, cyclosporine A, etoposide). Additional treatment modalities include immunoglobulins, anti-thymocyte globulin (ATG), interleukin-1 antagonist (anakinra) and monoclonal antibodies against CD52 (alemtuzumab), interleukin-6 (tocilizumab), TNF-α (infliximab) and CD20 (rituximab, in EBV triggered HLH) [2, 3, 8]. Interestingly, many patients of our series responded to dexamethasone monotherapy without any kind of cytotoxic agent. This is in contrast to the reported high rate of patients with M-HLH requiring more aggressive treatment [8] and highlights once more the differences between malignancy-induced and chemotherapy-associated HLH.

Observed case fatality rate significantly decreased in our cohort from 66.5 to 6.25%. This is partly due to improvements of treatment modalities, but might also be attributable to higher awareness of this complication leading to earlier initiation of specific and effective treatment. In addition, higher awareness might have led to detection of even milder cases with better prognosis in recent years. This is also reflected by the higher number of diagnosed cases in recent years (even if this difference is statistically not significant).

Fever was the leading symptom and was present in all patients. In haemato-/oncologic patients, persistent fever without source and not responding to empiric antimicrobial therapy should lead to the suspicion of HLH. Ferritin and sIL-2R serve as good markers to substantiate this suspicion [2, 14]. They were elevated in all patients even in the beginning of the respective episode. Without awareness of HLH during chemotherapy, this condition might be misinterpreted as fulminant bacterial sepsis [10]. Without HLH-specific treatment, patients might develop fatal multi organ failure.

Differentiation between viral triggered HLH and severe bacterial infection might be challenging, since inflammatory markers like CRP and PCT in nearly all patients were partly extensively elevated as commonly seen in bacterial infections. PCT is thought to be more specific for bacterial and possibly fungal infections [15–17] and has not been described in HLH, yet. In our cohort, however, both parameters were markedly elevated in patients with viral infections like HHV-6, HSV-1, JCV or RSV and even in patients without any documented infection. Therefore, neither CRP nor PCT seems to be able to discriminate between viral triggered HLH and severe bacterial infection. Furthermore, we found a correlation of laboratory parameters defining HLH (ferritin, sIL-2R, fibrinogen) in our cohort indicating a pathophysiologic interaction of these parameters. Interestingly, also CRP and PCT showed correlations with ferritin values which might indicate that these inflammatory parameters are part of the cytokine pattern of HLH.

While invasive *Aspergillus* infection and toxoplasmosis were diagnosed in two and one patient, respectively, no bacterial infection was identified as possible trigger despite repeated routine blood culture collection during febrile episodes. In contrast, we found viral infections or re-activations in the majority of cases. The rate of identified pathogens increased with the number of tested pathogens during the observation period. Beside EBV and CMV, which are commonly described as triggers for HLH [2, 3, 8], we detected several other viruses in association with HLH. Possibly as a consequence of our more comprehensive viral diagnostics (especially in more recent years), we found BKV, HHV-6, PVB19 and HSV-1 in association with HLH. While BKV and HHV-6 were yet reported only in single cases of HLH [18, 19], we detected these two viruses in about one third of patients. Detection rates in blood specimens for ADV, BKV, CMV, EBV, HHV-6 and PVB19 were significantly higher during HLH than in specimens from patients without HLH, while differences for BK viruria barely missed significance. In addition, several patients were diagnosed with multiple viral infections during HLH.

While our data clearly prove an association between viral re-/activation and HLH, we can only speculate on the causative properties of these viruses. However, in parallel to EBV and CMV which are commonly accepted as triggers for HLH, it seems likely that the other observed viruses may also play a causative role in the development of HLH.

In addition, total parenteral nutrition (TPN) containing lipids has been described as possible trigger of HLH [6]. In parallel, it might be speculated that liposomal amphotericin B (LAmB, AmBisome®) might also play a role as a trigger. In our cohort, the majority of patients received TPN, AmBisome® or both preceding the onset of HLH. Due to the descriptive character of our data, we cannot draw any conclusions on a possible pathophysiologic impact of these lipid-containing infusions and the high proportion of patients receiving TPN and/or LAmB might rather reflect the high rate of severely ill patients in our cohort.

## Conclusion

HLH is a condition of severe immune dysregulation with varying causes and clinical presentations. Chemotherapy-induced HLH should clearly be differentiated from malignancy-induced HLH. Patients with AML are more frequently affected than patients with ALL. Not only EBV and CMV but also other viruses including BKV, HHV-6 and PVB19 might serve as a possible trigger in immunocompromised patients. Clinical course might be fatal despite intensive treatment. On the other hand, timely and efficient immunosuppressive treatment leads to resolution of symptoms in many cases (despite documented infection). In many cases, corticosteroid monotherapy is sufficient. Awareness of this condition is important and fever without focus should prompt to consider this diagnosis. Elevated ferritin seems to be a good marker, while inflammatory markers like CRP and PCT are not useful to discriminate viral triggered HLH from severe bacterial infection.
